# Functionalized cerium oxide nanoparticles mitigate the oxidative stress and pro-inflammatory activity associated to the portal vein endothelium of cirrhotic rats

**DOI:** 10.1371/journal.pone.0218716

**Published:** 2019-06-24

**Authors:** Jordi Ribera, Juan Rodríguez-Vita, Bernat Cordoba, Irene Portolés, Gregori Casals, Eudald Casals, Wladimiro Jiménez, Victor Puntes, Manuel Morales-Ruiz

**Affiliations:** 1 Biochemistry and Molecular Genetics Department, Hospital Clínic of Barcelona, Institut d'Investigacions Biomèdiques August Pi i Sunyer (IDIBAPS), Centro de Investigación Biomédica en Red de Enfermedades Hepáticas y Digestivas (CIBERehd), Barcelona, Spain; 2 German Cancer Research Center, Heidelberg, Germany; 3 Catalan Institute of Nanotechnology (ICN), Bellaterra, Spain; 4 Department of Biomedicine-Biochemistry Unit, School of Medicine University of Barcelona, Barcelona, Spain; University of Navarra School of Medicine and Center for Applied Medical Research (CIMA), SPAIN

## Abstract

**Background and aims:**

The occurrence of endothelial alterations in the liver and in the splanchnic vasculature of cirrhotic patients and experimental models of liver diseases has been demonstrated. However, the pathological role of the portal vein endothelium in this clinical context is scarcely studied and, therefore, deserves attention. In this context, we aimed to investigate whether pathological endothelial activation occurs in the portal vein of cirrhotic rats.

**Methods:**

Cirrhosis was induced in wistar rats by CCl_4_ inhalation. We generated immortalized endothelial cells from the portal vein of control (CT-iPVEC) and cirrhotic rats (CH-iPVEC) by retroviral transduction of the SV40 T antigen. We assessed differential gene expression and intracellular reactive oxygen species (ROS) levels in iPVECs and in portal veins of control and cirrhotic rats. Finally, we assessed the therapeutic effectiveness of cerium oxide nanoparticles (CeO_2_NP) on reversing PVEC activation and macrophage polarization.

**Results:**

CH-iPVECs overexpressed collagen-I, endothelin-1, TIMP-1, TIMP-2, IL-6 and PlGF genes. These results were consistent with the differential expression showed by whole portal veins from cirrhotic rats. In addition, CH-iPVECs showed a significant increase in intracellular ROS and the capacity of potentiating M1 polarization in macrophages. The treatment of CH-iPVECs with CeO_2_NPs blocked intracellular ROS formation and IL-6 and TIMP-2 gene overexpression. In agreement with the *in vitro* results, the chronic treatment of cirrhotic rats with CeO_2_NPs also resulted in the blockade of both ROS formation and IL-6 gene overexpression in whole portal veins.

**Conclusions:**

Endothelial cells from portal vein of cirrhotic rats depicted an abnormal phenotype characterized by a differential gene expression and the induction of M1 polarization in macrophages. We identified the excess of intracellular reactive oxygen species (ROS) as a major contributor to this altered phenotype. In addition, we demonstrated the utility of the nanomaterial cerium oxide as an effective antioxidant capable of reverse some of these pathological features associated with the portal vein in the cirrhosis condition.

## Introduction

The portal vein carries blood from the gastrointestinal track to the liver contributing to the ~70% of the hepatic blood volume. This anatomical arrangement causes that the concentrations of certain hormones, metabolites and in some cases toxics xenobiotics are comparatively higher in the hepatic portal circulation than in any other vascular territory [1,2]. The exposure of the portal vein to this portal circulatory environment may result in the vascular activation of proinflammatory immune modulators that eventually may contribute to the development or worsening of portal hypertensive syndromes. For instance, the diseases most commonly encountered in the group of non-cirrhotic portal hypertensive syndromes are idiopathic portal hypertension and extrahepatic portal vein thrombosis and although the etiology of both pathologies are unclear it is considered to be pre-hepatic and vascular in origin with a significant pro-inflammatory component [3,4]. The pathological role of the portal vein in cirrhotic portal hypertensive syndromes has also been described in some contexts such as alcoholic cirrhosis. The resistance to portal blood flow in these patients is predominantly sinusoidal, but vascular remodeling of the portal vein also contribute to the overall intrahepatic resistance [5,6]. Based on these observations, we hypothesized that alterations in the functional properties of the portal vein may occur in portal hypertensive syndrome and that the pathophysiological characterization of this vascular bed is clinically relevant.

A normofunctional endothelium is crucial for the maintenance of vascular integrity through its anti-coagulant, anti-inflammatory and vasodilatory properties. However, several studies showed that the vascular endothelium may change to an activated/dysfunctional phenotype characterized by its pro-coagulant, pro-inflammatory and vasoconstrictive properties [7–11]. Endothelial cell activation has been documented in liver sinusoids in the context of chronic liver diseases [12–17]. However, very few publications have investigated the pathological role of the endothelial cell activation in other vascular areas such as the portal vein. A key limitation of this research field is the lack of established cell lines and the low-throughput characteristics of the classical cell isolation methods used to isolate endothelial cells from this vascular territory. In our study, we overcome this limitation by isolating primary endothelial cells from the portal vein of control and cirrhotic rats and immortalizing them by retroviral transduction of the SV40 large T antigen (iPVEC). The objectives of our study were 1) to characterize the differential phenotype of iPVECs after the establishment of these cell lines, 2) to correlate the cellular *in vitro* findings with the *in vivo* abnormalities of the portal vein in control and in cirrhotic rats and 3) to identify key molecular mechanisms responsible for switching the endothelium of the portal vein from a normofunctional to a dysfunctional state.

Here we show that endothelial cells from portal vein of CH rats depicted an abnormal phenotype characterized by a differential gene expression of proinflammatory, vasoactive and extracellular matrix remodeling genes. In addition, these cells showed a differential secretome and the capacity of potentiating M1 polarization in macrophages. We identified intracellular oxidative stress as a major contributor to this altered phenotype and evaluated the therapeutic utility of cerium oxide nanoparticles to revert the abnormalities found in the portal vein endothelium of cirrhotic rats.

## Materials and methods

### Experimental model of fibrotic rats

This study was performed in male adult Wistar rats with liver fibrosis (CCl_4_-treated rats) and control (CT) Wistar rats (Charles–River, Saint Aubin les Elseuf, France). Liver fibrosis was induced by inhalation of CCl_4_, as has previously been described [18]. All the animals were housed in 595×380×200 mm cages (Techniplast UK, 1354G Eurostandard Type IV) and were kept under constant temperature and humidity in a 12-h controlled dark/light cycle. The rats were fed *ad libitum* on a standard pellet diet. Control rats and CCl_4_-treated rats were studied 20 weeks after the start of the fibrosis induction protocol. During housing, animals were monitored twice daily for health status. No adverse events were observed. All animal procedures were approved by the Investigation and Ethics Committee of Animal Experimentation of the University of Barcelona.

### Isolation and immortalization of PVECs

Portal veins were isolated and digested with a 0.25% solution of collagenase A (Roche, Mannheim, Germany). Primary PVECs were isolated from the portal vein of control and cirrhotic (CH) rats using a rabbit anti-rat CD31 antibody (Abbiotec, San Diego, CA, USA) and magnetic beads coupled with a goat anti-rabbit secondary antibody (MACS system, Miltenyi Biotec, Germany). The immortalization of the endothelial cells was performed when the cells reached 75% of confluence by retroviral transduction of the middle T antigen from the SV40 virus and a geneticin resistance gene. After retroviral transduction, cells were maintained in Endothelial Cell Growth Medium 2 (Promocell, Heidelberg, Germany) supplemented with 50 U/mL penicillin, 50 μg/mL streptomycin and 10% fetal calf serum (FBS).

### Flow cytometry

Purity of endothelial cells was assessed by flow cytometry. First, cells were washed and re-suspended in PBS supplemented with 5% heat-inactivated FBS. A rabbit anti-rat CD31 (Abbiotec, San Diego, CA, USA) was used as a primary antibody. Then, cells were labeled using Alexa-488 conjugated anti-Rabbit secondary antibody (Life Technologies, Carlsbad, CA, USA). Positive cells were detected on a FACSCanto II flow cytometer (BD Biosciences, San Jose, CA, USA) and analyzed using FACSdiva software (BD Biosciences, San Jose, CA, USA). The negative population for the CD31 antigen was chosen from cells quantified in the absence of anti-CD31 antibody.

### Characterization and quantification of CeO_2_NP

CeO_2_NPs, with an average diameter of 4–20 nm, were synthesized by the chemical precipitation of cerium (III) nitrate hexahydrated (Sigma-Aldrich, St. Louis, MO, USA) as previously described [19,20]. High resolution Transmission Electron Microscopy (HR-TEM) was performed using a JEOL-1010 TEM (JEOL, Tokyo, Japan) with the field-emission gun operating at 80 kV. Prior to the analysis, 15μL from the nanoparticles solution were dispersed on a copper grid coated with a formvar film. The samples were then let to dry for TEM observation and digital photomicrographs were taken (BioScan Gatan, CA, USA). Cerium concentration in tissue was measured by inductively coupled plasma mass spectrometry (ICP-MS, Agilent 7500; Agilent Technologies CA, USA). The quantification is performed by interpolation in a standard curve obtained from a commercial 1000 ppm Ce standard (Sigma-Aldrich, St. Louis, Missuori, USA).

### *In vivo* and *in vitro* studies with CeO_2_NPs treatment

For the *in vivo* study, control and cirrhotic rats were randomly distributed into two groups. One group was treated with 0.1 mg/kg of CeO_2_NPs in TmaOH 0.8mM (n = 20 for each experimental condition) and CCl_4_ treatment was maintained thereafter. The second group received vehicle (saline solution containing TMAOH ammonium salts 0.8 mM) (n = 20 for each experimental condition). CeO_2_NPs were dispersed in saline solution and intravenously given as a bolus (500 μL) through the tail vein. For the intravenous injection, animals were initially anaesthetized under a 5% of isoflurane inhalation and then maintained under 2–2.5% isoflurane for the rest of the procedure, with an oxygen flow rate of 800 cc/min. The treatments were administered twice per week for two weeks. At the end of the treatments, rats were euthanized by anaesthesia overdose (5% of isoflurane inhalation in oxygen) and organs dissected and kept at -80 ºC for further analysis.

For the *in vitro* study, cells were treated with CeO_2_NPs (1μg/mL) for 24 hours in a water-jacketed CO_2_ incubator (Forma Scientific Series II, 3131, Marietta, OH).

### Scanning electron microscope (SEM)

Portal vein samples were fixed in 2% paraformaldehyde and 2.5% gluteraldehyde for further ultrastructural examination. Next, each sample was ion-sputter-coated and observed at low electron power microscope to increase contrast in a Jeol JSM 5600 LV scanning electron microscope (Jeol Ltd., Tokyo, Japan) equipped with a BioScan camera (Gatan, CA, USA), and digital photomicrographs were taken.

### Macrophage differentiation

Briefly, femurs from rats aged 8 to 10 weeks were flushed and bone marrow derived cells were collected by centrifugation at 450×g for 5 min at 4°C. Cells were then re-suspended in DMEM, supplemented with 10% heat-inactivated FBS and 30% of L-929 M-CSF conditioned medium, and cultured at a density of 1×10^6^ cells/mL in non-tissue culture treated plastic dishes (BD Biosciences, San Jose, CA, USA) in a humidified atmosphere (95% air, 5%CO_2_) at 37°C. Medium was replaced every 2 days. After 6 days, adherent cells were collected and cell viability was measured by trypan blue. Bone marrow derived macrophages (BMDM) were then diluted in DMEM containing 10% of heat-inactivated FBS and plated in culture dishes for further experiments.

### Generation of M-CSF containing conditioned medium

M-CSF overexpressing L929 cells were kindly donated by Prof. Antonio Celada (Macrophage Biology Group, School of Biology-University of Barcelona). Cells were cultured up to a 75% of confluence in DMEM supplemented with 2% of heat-inactivated FBS, 50 U/ml penicillin and 50 μg/ml streptomycin. After 5 days of culturing, medium was replaced keeping the collected medium. On the tenth day, the medium was collected and mixed with the conditioned medium obtained at day 5. After filtration with a 0.2 μm filter, the final conditioned medium was stored at -20ºC for later use. The concentration of M-CSF in this conditioned medium was 5 ng/mL, according to ELISA quantification (Cusabio, Baltimore, MD, USA).

### Immunocytofluorescence

The iPVECs were fixed with 4% paraformaldehyde for 10 minutes. Cells were washed three times with PBS and permeabilized with 0.1% Triton for 4 minutes at 4°C. Then, cells were incubated for 1 hour with rabbit anti-rat CD31 primary antibody (Abbiotec, San Diego, CA, USA) and anti-rabbit FITC secondary antibody (Life Technologies, Carlsbad, CA, USA) for 1 h. Immunofluorescence was visualized with a fluorescent microscope (Nikon Eclipse E600, Kawasaki, Kanagawa, Japan).

### Real-Time PCR

Total RNA was extracted from cultured PVEC using the Trizol reagent (Life Technologies, Carlsbad, CA, USA). One microgram of total RNA was reverse transcribed using First Strand cDNA Synthesis Kit (Roche, Mannheim, Germany). Subsequently, complementary DNA samples were amplified for 30–35 cycles (94 for 30 seconds, 55–60°C for 30 seconds, and 72 during 1 minute; LigthCycler 480-Roche Diagnostics). To normalize the results, HPRT gene was used as reference. Specific primers for amplification of the complementary DNA were:

Collagen I, 5'-AGACCTGGCGAGAGAGGAGT-3' (forward primer) and 5'-ATCCAGACCGTTGTGTCCTC-3' (reverse primer);

TIMP1, 5’-CATGGAGAGCCTCTGTGGAT-3' (forward primer) and 5'-TGTGCAAATTTCCGTTCCTT-3' (reverse primer);

TIMP2, 5’-GACAAGGACATCGAATTTATCTACAC-3' (forward primer) and 5'-CCATCTCCTTCCGCCTTC-3' (reverse primer);

PlGF, 5’-GTTGGCTGTGCACTCCCAG-3' (forward primer) and 5'-GTTGGCTGTGCACTCCCAG-3' (reverse primer);

Endothelin-1, 5'-CTCCTCCTTGATGGACAAGG-3' (forward primer) and 5'-CTTGATGCTGTTGCTGATGG-3' (reverse primer);

IL-6, 5'-GCCCTTCAGGAACAGCTATGA-3' (forward primer) and 5'-TGTCAACAACATCAGTCCCAAGA-3' (reverse primer);

MMP2, 5’-GCGCTTTTCTCGAATCCAT-3' (forward primer) and 5'-GGGTATCCATCTCCATGCTC-3' (reverse primer);

eNOS, 5’-TCTACCGGGACGAGGTACTG-3' (forward primer) and 5'-CTGTCCTCAGGAGGTCTTGC-3' (reverse primer);

iNOS, 5’-ACAACAGGAACCTACCAGCTCA-3' (forward primer) and 5'-GATGTTGTAGCGCTGTGTGTCA-3' (reverse primer);

TNF-α, 5’-CAAGGAGGAGAAGTTCCCAA-3' (forward primer) and 5'-CTCTGCTTGGTGGTTTGCTA-3' (reverse primer);

Arginase-1, 5’-CAAGCTGGGAATTGGCAAAG-3' (forward primer) and 5'-GGTCCAGTCCATCAACATCAAA-3' (reverse primer); and

HPRT, 5’-GACCGGTTCTGTCATGTCG-3' (forward primer) and 5'-ACCTGGTTCATCATCACTAATCAC-3' (reverse primer).

### Characterization of iPVEC secretome by mass spectrometry

Protein profiling was performed by surface-enhanced laser desorption/ionization time-of-flight mass spectrometry (SELDI-TOF-MS) using the eight-spot format CM10 ProteinChip arrays (Bio-Rad, Hercules, CA, USA). Conditioned cell culture media from iPVECs was loaded onto CM10 Protein Chip arrays. Prior to sample loading, spots were equilibrated two times with 200 μL of CM binding/washing buffer (0.1 M sodium acetate, pH 4). Each sample was loaded in duplicate randomly in order to minimize any systematic error. Forty microliters of conditioned medium were incubated in 60 μL of CM binding buffer for 30 minutes on a shaker at room temperature. Afterwards, arrays were washed three times with 200 μL CM washing buffer for 5 minutes at room temperature. Unbound serum proteins were removed by washing twice with deionized water. Thereafter, arrays were air-dried and 1 μL of energy-absorbing matrix (saturated sinapinic acid in an aqueous solution containing 50% acetonitrile and 0.5% TFA) was added twice to each spot. The surface was allowed to air dry between each application. The array was read by using the ProteinChip PBS II reader (BioRad, Hercules, CA, USA). Each spot was read at 3000 nJ energy laser intensity. All spectra were calibrated using two external calibration standards (all-in-one peptide standard and all-in-one protein standard, BioRad, Hercules, USA). A peak resolution was optimized within 5.000 m/z, 12.000 m/z or 19.000 m/z according to low, medium or high energy laser intensity, respectively.

### Western blot experiments

Cell lysates were prepared in a lysis buffer (Tris–HCl 20 mM pH 7.4 containing 1% Triton X-100, 0.1% SDS, 50 mM NaCl, 2.5 mM EDTA, 1 mM Na_4_P_2_O_7_ 10H_2_O, 20 mM NaF, 1 mM Na_3_VO_4_, 2 mM Pefabloc and Complete from Roche). Proteins were separated on a 7.5% SDS-polyacrylamide gel (Mini Protean III, BioRad, Richmond, Ca) and transferred for 2 hours at 4ºC to nitrocellulose membrane (Transblot Transfer Medium, BioRad, Richmond, CA) that was stained with Ponceau-S red as a control for protein loading. The membrane were incubated at 4ºC with rabbit polyclonal anti-rat VE-Cadherin (Abcam plc, Cambridge, UK) overnight in a 1:1000 dilution. Then, the membrane was incubated with goat anti-rabbit peroxidase-conjugated secondary antibody at a 1:5000 dilution (Cell Signaling, Beverly, MA) for 1 hour at room temperature. The bands were visualized by chemiluminescence (ECL western blotting analysis system; Amersham Biosciences).

### Reactive oxygen species measurement

Fluorescence spectrophotometry was used to measure ROS, with 2’,7’-DCF-DA as the probe (Life Technologies, Carlsbad, CA, USA). DCF-DA readily diffuses through the membrane and is enzymatically hydrolyzed by intracellular esterases to the nonfluorescent DCFH, which can then be rapidly oxidized to fluorescent DCF in the presence of ROS. PVECs incubated in the presence of CeO2NPs or vehicle were washed with PBS buffer and incubated with 10 μM DCF-DA in DMEM for 50 min at 37 ºC in the dark. The supernatant was collected to measure the extracellular production of ROS, and the intensity of fluorescence was immediately read in a fluorescence spectrophotometer (FLUOstar OPTIMA; BMG LABTECH, Ortenberg, Germany) at 485 nm for excitation and at 520 nm for emission.

Oxidative stress present in rat portal veins was determined by malondialdehyde (MDA) assay (ELISA) according manufacturer’s instructions (Cell Biolabs, San Diego, USA).

### Statistical analysis

Quantitative data were analyzed using GraphPad Prism, version 5 (GraphPad Software, Inc., San Diego, CA) and public libraries from The Comprehensive R Archive Network (CRAN; http://CRAN.R-project.org) rooted in the open source statistical computing environment R, version 3.4 (http://www.R-project.org/). The statistical analysis of the results was performed using unpaired Student's t-tests and ANOVA models (with Tukey's post hoc test) with normally distributed data. For other type of data, the Mann–Whitney U-test and the Kruskal-Wallis tests (with Dunn post hoc test) were used. Results are expressed as mean±s.e.m and considered significant at a p value lower than 0.05.

## Results

### Generation and characterization of immortalized PVECs from portal vein of control and cirrhotic rats

**Fig 1:**
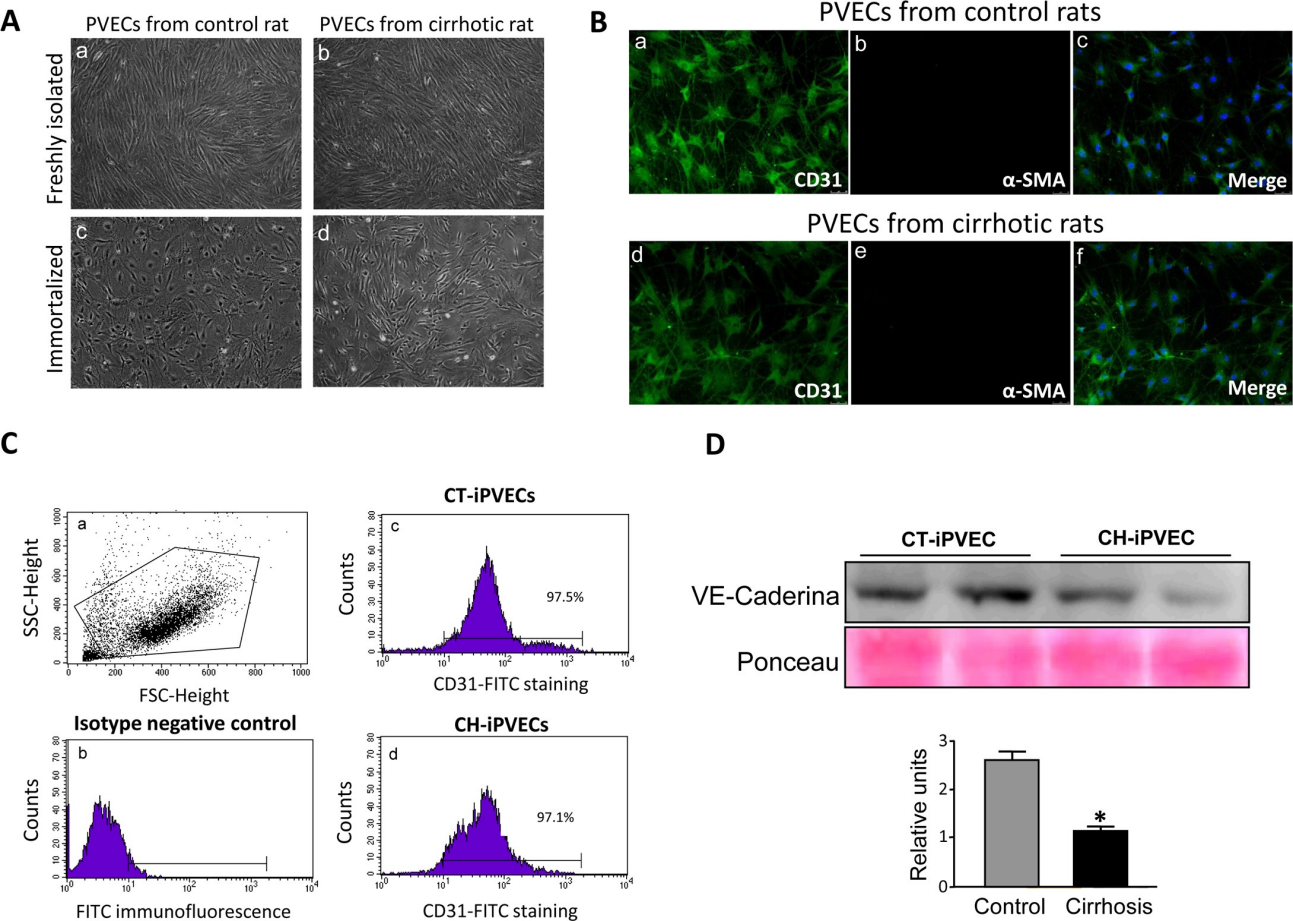
Immortalized PVEC maintain endothelial cell features. PVECs were isolated from portal veins from control and cirrhotic rats. Immortalization of the cells was carried out using a retrovirus long sequence containing the SV40 virus T antigen. **A** Images taken by optical microscope of PVECs from control (left) and cirrhotic (right) rats, before (top) and after (bottom) immortalization, are shown. **B** CD31 and α-SMA immunostainings are shown for iPVECS established from control (a, b and c panels) and cirrhotic rats (d, e and f panels). The merged panels show CD31 and α-SMA colocalization. Nuclei were stained with DAPI (blue) (n = 5). Original magnification 200X. **C** iPVECs were immunostained for CD31 and analyzed by flow cytometer. Panel **a** shows dot-blot graph of the cell population. The negative population for the CD31 antigen was chosen from cells quantified in the absence of anti-CD31 antibody (panel c), CT-iPVEC (panel b), and CH-iPVEC (panel d) (n = 5). **D** cell lysates were analyzed by western blot. Upper panel shows VE-Cadherin immunoblot with loading control by ponceau staining. Lower panel shows densitometric analysis of the western blot. *p<0.*05* vs control.

To investigate the cellular mechanisms that contribute to the portal vascular dysfunction characteristic of cirrhosis, we isolated rat portal endothelial cells (PVEC) from control and cirrhotic rats. After the immortalization procedure using middle T antigen we established two cell clones for each experimental condition. Next, cells were tested for the maintenance of endothelial cell markers. Freshly isolated PVEC maintained the characteristic cobblestone morphology of endothelial cells (Fig 1A, panels a and b). Immortalization resulted in altered cell shape and a lack of the directional alignment characteristic of the primary PVEC (Fig 1A, panels c and d). Despite the change in morphology, nearly 97% of immortalized iPVEC (iPVEC) conserved the expression of the endothelial cell markers CD31 and VE-cadherin, as assessed by immunofluorescence (Fig 1B), flow cytometry (Fig 1C) and western blot (Fig 1D). In the case of VE-cadherin, iPVEC from cirrhotic rats expressed lower levels of VE-cadherin compared with control rats, suggesting that these cells have maintained part of the altered phenotype caused by the cirrhosis condition. Alpha-smooth muscle actin (α-SMA) protein was undetectable in either of the immortalized iPVEC clones established from control and cirrhotic rats (Fig 1B, panels b and e), ruling out the presence of contaminating pericytes or vascular smooth muscle cells.

### iPVEC and portal veins from cirrhotic rats showed a similar gene expression profile and increased levels of ROS

**Fig 2:**
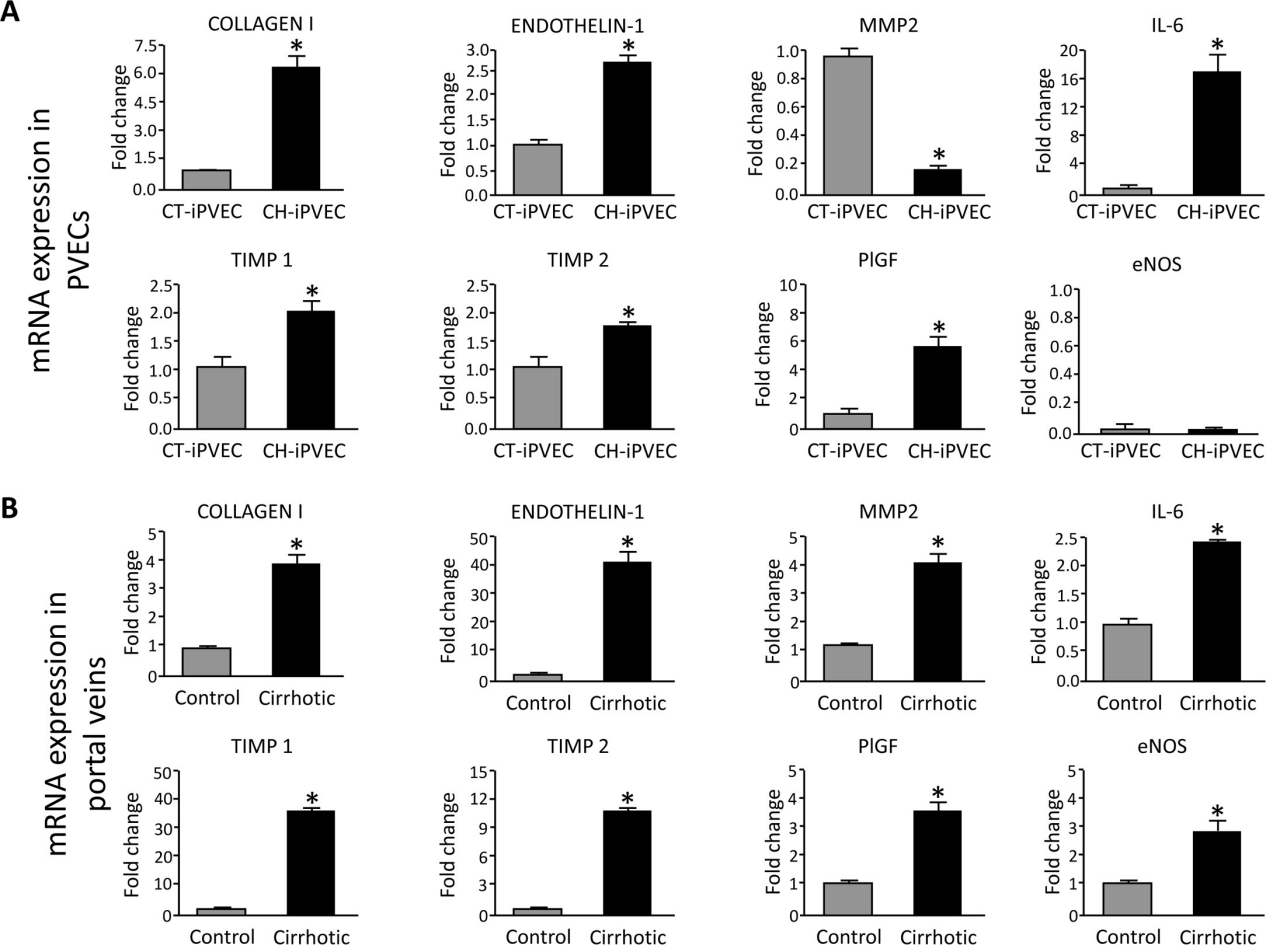
Comparison of gene expression profiles between CT-iPVEC and CH-iPVEC. **A** iPVECs established from the portal vein of control (CT-iPVEC) or cirrhotic rats (CH-iPVEC) were lysed in trizol and their mRNA expression was analyzed by real-time PCR, as described in materials and methods. Graph show the different expression levels for the corresponding genes. mRNA levels are illustrated as fold change relative to HPRT mRNA levels. *p<0.*05* vs control (n = 10). **B** Portal veins from either control or cirrhotic rats were extracted and Lysed in Trizol. mRNA expression was analyzed by real-time PCR. Graph show the different expression levels for the corresponding genes. mRNA levels are illustrated as fold change relative to HPRT mRNA levels. *p<0.*05* vs control (n = 10).

To further investigate the possibility that iPVECs from cirrhotic rats maintained part of the altered phenotype caused by the cirrhosis condition, we measured by real-time PCR the expression of genes that were previously described to be differentially expressed in other vascular territories due to chronic liver disease [17,18,21,22]. All of the genes that we measured in iPVECs have known roles in inflammation (IL-6), regulation of the vascular tone and remodeling (collagen 1, endothelin-1, MMP2, TIMP1, TIMP2 and eNOS). Col1A1, endothelin-1, MMP2, IL-6, TIMP1, TIMP2 and PlGF were differentially expressed in iPVECs from cirrhotic rats (Fig 2A). All of them except MMP2 were up-regulated in the cirrhosis condition being IL-6 the gene showing the largest extent of up-regulation. In the case of MMP2, iPVECs from cirrhotic rats showed a 5-fold downregulation of this gene compared with iPVECs from control rats (Fig 2A). Unexpectedly, negligible expression of eNOS was seen in the iPVECs clones obtained from control or cirrhotic rats.

To study the *in vivo* correlation of the differential expression found in iPVECs, portal veins from control and cirrhotic rats were processed to quantify mRNA expression. The same panel of genes was measured in whole portal vein and the result was compared with that obtained in iPVECs. As shown in Fig 2B, portal vein from cirrhotic rats overexpressed collagen I, endothelin-1, IL-6, TIMP1, TIMP2 and PlGF. This profile of overexpressed genes is consistent with the differential expression found in iPVEC from cirrhotic rats except for MMP2, which showed opposite mRNA changes in portal vein (~4-fold overexpression) and iPVECs (~5-fold downregulation), compared with the control condition. The eNOS gene was also found to be overexpressed in the portal vein of cirrhotic rats, in agreement with what have been described in other vascular areas studied in the CCl_4_, BDL or PVL experimental rat models [2].

**Fig 3:**
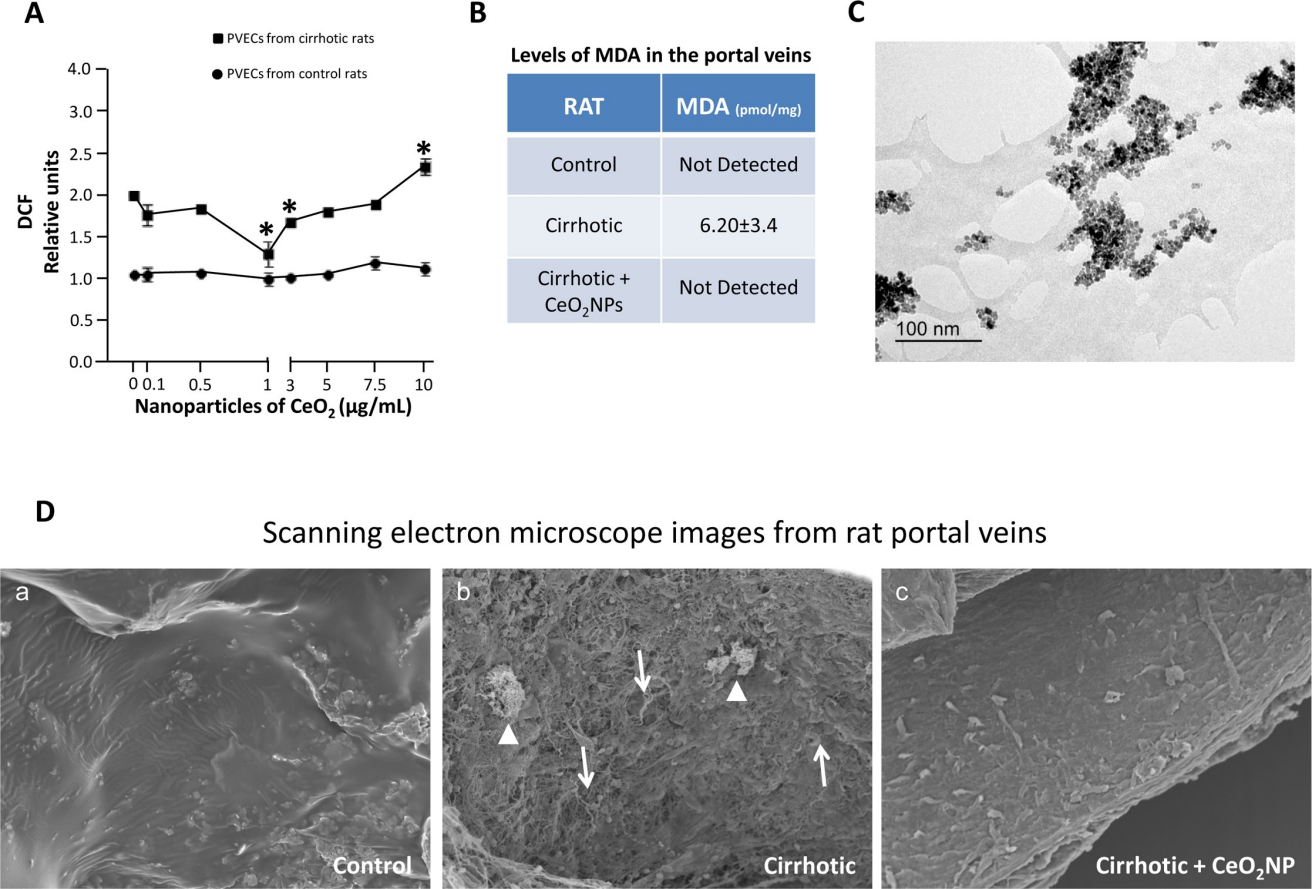
Increased levels of ROS in CH-PVEC and portal vein of cirrhotic rats are reduced by CeO_2_NPs treatment. **A** HR-TEM photomicrograph of CeO_2_NPs (400,000X). The suspension of CeO_2_NPs for HR-TEM was dispersed on a copper grid coated with a formvar film as described in material and methods. **B** oxidative stress was measured in cell lines using DCF. Graph shows the oxidation level at basal and different concentrations of CeO_2_NPs. *p<0.*05* vs basal condition (n = 10). **C** oxidative stress was measured in portal veins using MDA assay. Table shows the oxidation level expressed as pmol of MDA per mg of portal vein tissue extracted from rats treated with vehicle or CeO_2_NPs (0.1 mg/kg) (n = 5). **D** CeO_2_NP treatment promoted angioarchitecture normalization in the portal vein of cirrhotic rats. Representative scanning electron micrographs of the vascular endothelium from portal veins of control, cirrhotic and CeO_2_NPs treated cirrhotic rats (X 500; 15.0 kV). Arrows denote high profusion of endothelial exfoliation with loss of cell-cell junctions. Arrow heads denote extra-vascular cell adhesion to the vascular endothelium (n = 3).

It has been established that vascular dysfunction is closely associated with elevated production of vascular ROS. For instance, oxidative stress up-regulates the expression of endothelin-1 [23], pro-inflammatory mediators such as IL-6 [24] and vascular remodeling factors such as collagen 1 [25,26], which we found overexpressed in both iPVEC and portal vein from cirrhotic rats. Therefore, it is possible that vascular oxidative stress may be involved in the phenotypic switch that we observed in endothelial cells from portal veins of cirrhotic rats. In order to verify this hypothesis, we investigated the production of reactive oxygen species in iPVEC from both control and cirrhotic rats. We added dihydrodichlorofluorescein diacetate (H_2_DCF-DA) to cells in culture to measure oxidative stress in iPVEC. H_2_DCF-DA accumulates preferentially in the cytosol and the intracellular oxidation of H_2_DCF into the fluorochrome DCF is measured to evaluate generalized oxidative stress. We compared the baseline level of oxidative stress in both groups of iPVECs. As shown in Fig 3A, DCF levels were 2-fold higher in iPVEC from cirrhotic rats compared with iPVEC isolated from control rats. Oxidative stress was also measured in whole portal veins from cirrhotic and control rats by malondialdehyde (MDA) quantification. In agreement with the ROS measurement in iPVECs, MDA levels were between 3 and 6-fold higher in portal veins from cirrhotic rats compared with portal veins from control rats (Fig 3B).

### CeO_2_NPs treatment reduced oxidative stress of portal vein endothelial cells *in vitro* and *in vivo*, and partially normalizes the endothelium angioarchitecture in the portal vein

An important question is whether and effective anti-oxidant intervention in the portal vein may result in a normofunctional vascular reprogrammation of the transcriptome in CCl_4_-treated rats. With this objective in mind we treated iPVEC and cirrhotic animals with the nanomaterial cerium oxide. Fig 3C shows the transmission electron microscopy (TEM) images of the CeO_2_NPs suspension, which is characterized by a similar polyhedral shape of the nanoparticles with loose agglomerates and an average diameter of 4–20 nm.

The antioxidant activity of CeO_2_NPs was assessed on iPVECs by measuring the intracellular accumulation of DCF. CeO_2_NPs tested in the dose range 0.1–10 μg/mL did not appear to produce any change on DCF values in iPVEC from control rats, suggesting that the baseline levels of ROS in the iPVECs from control rats are negligible. In contrast, CeO_2_NPs treatment of cirrhotic iPVEC reduces significantly the intracellular levels of ROS at the doses from 1 to 3 μg/mL. Specifically, CeO_2_NPs showed the highest scavenging activity at a concentration of 1 μg/mL. At this optimal concentration, DCF fluorescence significantly decreased, approaching the levels found in control iPVECs. However, cirrhotic iPVECs exposed to CeO_2_NPs at higher doses than 3 μg/mL showed a hormetic-like dose response characterized by an increasing-dose inefficacy in ROS scavenging (Fig 3A). The direct toxic effect of CeO_2_NPs on iPVECs was also evaluated with the Trypan Blue exclusion test. At the concentration tested (0.1–10 μg/mL), the exposure of the cells with the nanoparticles did not affect cell viability.

To translate our *in vitro* results to the *in vivo* situation, we investigate whether portal vein from cirrhotic rats reduced its oxidative stress levels in response to the chronic treatment with CeO_2_NPs. After 2 weeks of CeO_2_NPs treatment, portal veins from both control and cirrhotic rats retained a 7% of the total dose of nanoparticles given via tail vein injection, as measured by inductively coupled plasma mass spectrometry (14.26 μg from the total 200 μg CeO_2_NPs administered over the two-week treatment period). After the treatment, we measured the levels of MDA in the portal vein from cirrhotic rats treated with CeO_2_NPs and we found that the levels were negligible compared with portal vein from cirrhotic rats without treatment (Fig 3B, last row). These findings correlated with an improvement of the endothelium monolayer in the portal vein. Under scanning electron microscope (SEM), cirrhotic portal vein presents a rougher and unstructured surface (Fig 3D, panel b), while these abnormalities were partially corrected after the chronic treatment with CeO_2_NPs resulting in a smoother surface (Fig 3D, panel c), more similar to the control condition (Fig 3D, panel a).

### Conditioned medium from CH-iPVEC polarized macrophages to an M1 phenotype

**Fig 4:**
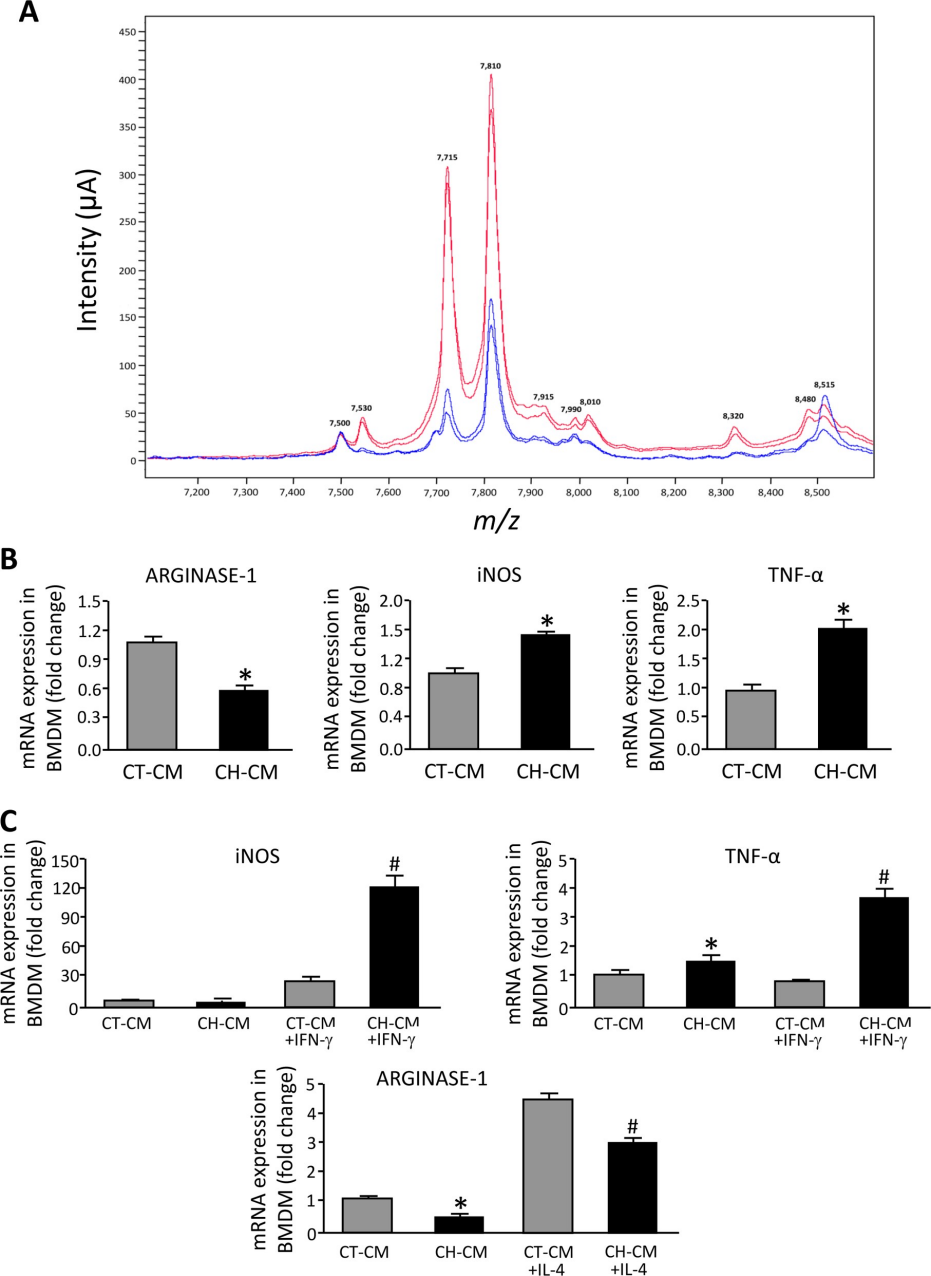
Characterization of iPVEC secretome and iPVEC-induced macrophage polarization. **A** Representative proteomic profile of conditioned cell culture media (DMEM supplemented with 2% FBS) obtained from CT-PVEC (blue spectrogram) and CH-PVEC (red spectrogram) after 3 days of cell culture. Graph shows a differential proteomic signature in the segment of the SELDI-TOF-MS spectra ranging from *m/z* 7,2 kDa to *m/z* 8,6 kDa (n = 3). **B** Bone marrow derived macrophages (BMDM) were incubated with conditioned medium from either CT-PVEC (CT-CM) or CH-PVEC (CH-CM). Panel shows gene expression levels for the different genes analyzed. mRNA levels are illustrated as fold change relative to HPRT mRNA levels *p<0.*01* vs CT-CM (n = 10). **C** BMDM were incubated with CT-CM or CH-CM and simultaneously with either IL-4 or IFN-γ for 8h (10ng/mL). Panels show iNOS and TNF-α gene expression after IFN-γ treatment and arginase-1 (Arg1) after IL-4 treatment. *p<0.*01 vs*. CT-CM, ^#^*p<0*.*01 vs*. CT-CM treated with IL-4 or INF-γ (n = 10).

The interaction between the endothelium and leukocytes is critical for the activation and the recruitment of inflammatory cells. We have found that iPVEC from cirrhotic rats overexpressed IL-6. Considering this proinflammatory phenotype, we next investigated whether the secretome of iPVECs from cirrhotic rats affected macrophage polarization and, therefore, inflammatory response. For this purpose, we collected conditioned medium of iPVEC from control and cirrhotic rats that were cultivated in presence of 2%FBS for 72 hours. The iPVEC secretomes from each cell clone isolated from the control and the cirrhosis condition were characterized by proteomics (SELDI-TOF mass spectrometry). As shown in Fig 4A, eight peptidic clusters from the collected conditioned medium of the two CH-iPVEC clones showed a significant higher intensity (*m/z* of 7.530, 7.715, 7.810, 7.915, 7.990, 8.010, 8.320 and 8.480 kDa), while 2 peaks (*m/z* of 7.500 and 8.515 kDa) showed a lower intensity when compared with the CT-iPVEC conditioned media. This divergence in the secretomes of iPVEC suggests that besides presenting a differential transcription reprogramming, iPVECs from cirrhotic rats may also interact differentially with their cellular environment through their secretome.

To determine the effect of iPVECs on macrophage polarization, we exposed bone marrow derived macrophages to conditioned medium from CT-iPVEC or CH-iPVEC for 8h. Without any additional stimuli, conditioned medium from CH-iPVEC induced the expression of iNOS and TNF-α in macrophages and downregulated arginase-1 (Arg-1) mRNA, compared with conditioned medium from CT-iPVEC (Fig 4B). To investigate further the effect of iPVEC on macrophage polarization induced by the cytokine environment, we characterized the effect of conditional medium on the induction of M1 and M2 polarization by IFN-γ and IL-4, respectively. As shown in Fig 4C, CH-iPVEC conditioned medium potentiates the effect of IFN-γ on the induction of iNOS and TNF-α overexpression in macrophages. Consistently, we found that macrophages exposed to CH-iPVEC showed a significant downregulation of arginase-1 mRNA after incubation with IL-4, compared with the CT-iPVEC treatment. These results together, demonstrate the ability of the CH-iPVEC secretome to polarize macrophages toward the M1 fate in both basal conditions and after cytokine stimulation.

### CeO_2_NPs treatment switch CH-iPVEC-induced macrophage polarization from M1 to M2

**Fig 5:**
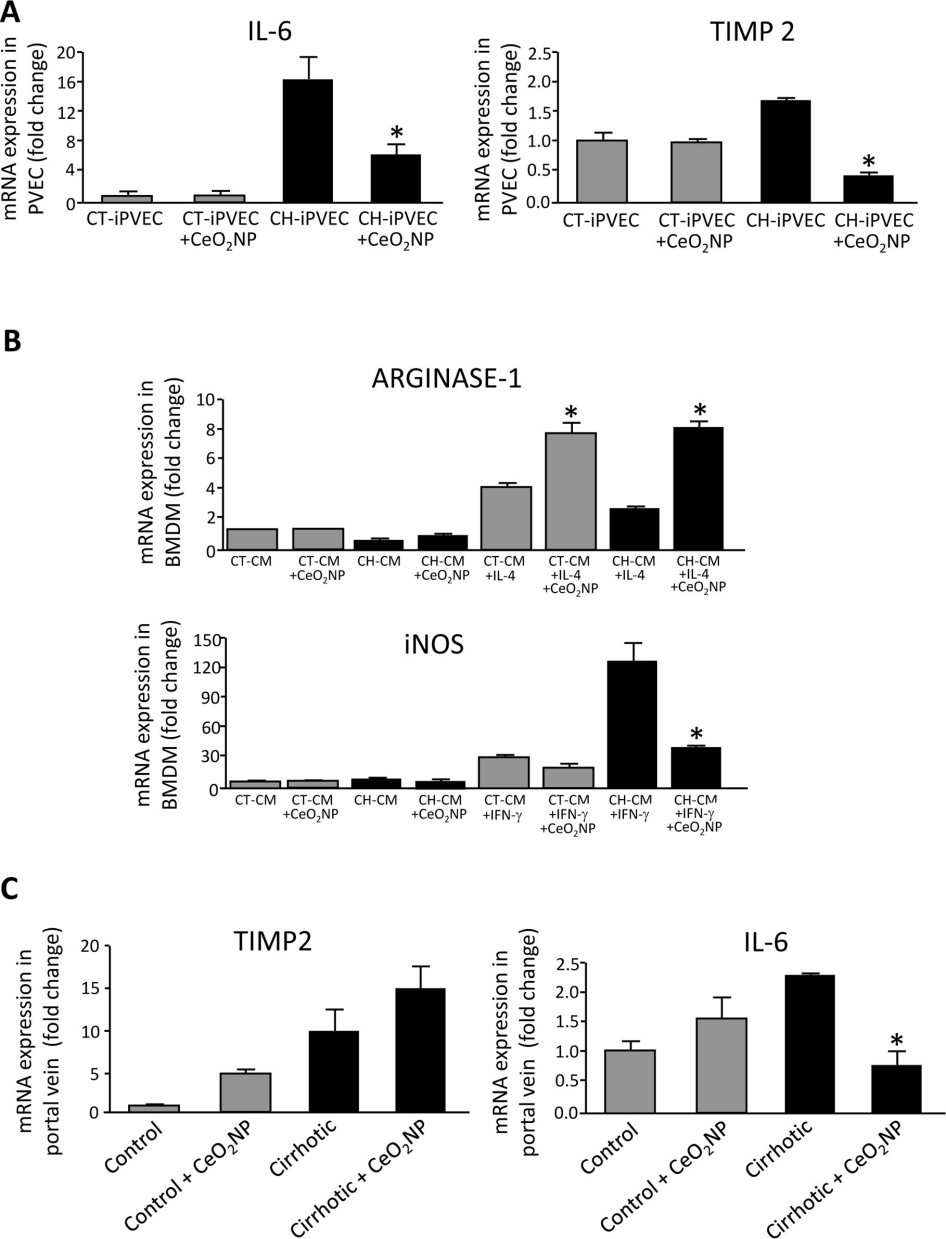
CeO_2_NPs treatment modified the altered gene expression pattern of CH-PVEC and portal vein from cirrhotic rats and potentiates macrophage M2 polarization. **A** CT-iPVECs and CH-iPVECs were incubated with vehicle or 1μg/mL CeO_2_NPs for 24h. Cells were lysed in trizol and mRNA levels of IL-6 (left graph) and TIMP2 (right graph) were quantified by real-time PCR. mRNA levels are illustrated as fold change relative to HPRT mRNA levels. *p<0.*05* vs CH-PVEC without CeO_2_NPs (n = 5). **B** BMDM were incubated with CT-CM or CH-CM from iPVEC treated with CeO_2_NPs (1μg/mL) for 24h or vehicle and stimulated simultaneously with either IL-4 or IFN-γ 10ng/mL for 8h. Top graph shows arginase-1 gene expression after IL-4 stimulation and bottom graph shows iNOS gene expression after IFN-γ stimulation. mRNA levels are illustrated as fold change relative to HPRT mRNA levels. *p<0.*05 vs*. their corresponding CeO_2_NPs-untreated control groups (n = 10). **C** Portal veins from either control or cirrhotic rats treated *i*.*v*. with vehicle or 0.1 mg/kg CeO_2_NPs for 2 weeks were extracted and lysed in trizol. mRNA expression was analyzed by real-time PCR and represented as fold change relative to HPRT mRNA levels. *p<0.*05 vs*. its corresponding CeO_2_NPs-untreated control group (n = 5).

Once we established the optimal concentration for CeO_2_NPs treatment, we next investigated whether the protection against oxidative stress in iPVEC improves the altered gene expression pattern that we previously described in the cirrhotic condition. The treatment of iPVECs with 1μg/mL CeO_2_NPs for 24 hours did not modify the expression of Col1A1, endothelin-1, TIMP1, PlGF or MMP2; compared with vehicle treatment (S1 Fig). Interestingly, CeO_2_NPs treatment caused a significant decrease in the expression of IL-6 and TIMP-2 in CH-iPVEC, suggesting that oxidative stress is mediating the overexpression of these genes (Fig 5A).

Next, we also investigate the effect of CeO_2_NPs on iPVEC-induced macrophage polarization. iPVEC were incubated with CeO_2_NPs for 24h. After medium removal, cells were placed in DMEM supplemented with 2% FCS for three additional days. Conditioned medium from CH-iPVEC treated with CeO_2_NPs significantly increased the levels of arginase-1 on macrophages in response to IL-4 treatment, compared with CH-iPVEC treated with vehicle (Fig 5B, top graph). Additionally, CeO_2_NPs treatment blocked iNOS overexpression induced by CH-iPVEC conditioned medium to a similar level to that obtained by the CT-iPVEC condition upon IFN-γ stimulation (Fig 5B, bottom graph). Therefore, CeO_2_NPs treatment potentiates macrophage M2 polarization through changes on the CH-iPVEC secretome.

### Cirrhotic rats treated chronically with CeO_2_NPs down-regulated IL-6 expression in portal vein

As showed above, CeO_2_NP treatment can modify the expression of certain genes in iPVECs *in vitro*. Therefore, we next assessed whether the chronic treatment of rats with CeO_2_NPs also has an effect on the portal vein through the modification of its gene expression pattern. To this aim, we quantified the expression of the same genes that were differentially expressed in CH-iPVECs after CeO_2_NPs treatment, IL-6 and TIMP-2. As shown in Fig 5C (right graph), TIMP-2 showed similar expression levels in the portal vein of control and cirrhotic rats regardless of the CeO_2_NPs treatment. Interestingly and in agreement with the *in vitro* experiments, the treatment of cirrhotic rats with CeO_2_NPs significantly downregulated IL-6 in portal vein, compared with vehicle treated rats (Fig 5C, left graph). These results demonstrated the *in vivo* utility of CeO_2_NPs treatment for modulating the pathological phenotype of the vasculature in chronic liver disease.

## Discussion

Our results describe for the first time that the endothelium of the portal vein from cirrhotic rats has pro-inflammatory properties, which are the overexpression of IL-6 and the induction of M1 macrophage polarization. We showed that an excess of ROS is responsible of this vascular alteration associated with cirrhosis because the treatment with the CeO_2_ antioxidant nanoparticles reverts this pro-inflammatory phenotype.

The term ROS includes free radicals and strong oxidants such as superoxide anion radical (·O_2_^-^), hydroxyl radical (HO·), hydrogen peroxide (H_2_O_2_) and hypochlorous acid (HOCl), all of them being highly reactive. When ROS levels exceed cellular antioxidant defenses oxidative damage develops within the cells promoting molecular damage in DNA, lipids and proteins. The presence of oxidative stress has been described in most of the clinical conditions associated with liver fibrosis and portal hypertension as well as in experimental models of liver injury (NASH, HCV, alcoholic liver cirrhosis, hemochromatosis, Wilson's disease, primary biliary cirrhosis, cholestasis, cirrhosis induced by CCl_4_ in rodents, and bile duct ligation in rodents). Concurrently, the association between high levels of oxidative stress and a reduction of antioxidant defenses has also been reported in the same or similar pathological situations [27,28]. Several strategies have been investigated to diminish ROS damage such as the transduction of the liver of cirrhotic rats with adenovirus encoding for SOD, which resulted in a significant improvement of portal pressure [29]. In the same experimental model, hesperidin -an antioxidant bioflavonoid- also ameliorates liver damage through the inhibition of oxidative stress [30]. In addition, Bataller et al. demonstrated that NADPH oxidase is present in activated HSCs and generate ROS in an angiotensin II dependent way. The link between NADPH oxidase activity and fibrosis was further demonstrated *in vivo* in mice lacking a functional NADPH oxidase [31]. All these studies highlight the need of targeting hepatic oxidative stress in cirrhotic livers. However, studies investigating the pathological role plaid by ROS in the portal vein of patients or animal models with chronic liver disease are still lacking.

In other pathological contexts, several authors have described the vascular endothelium as one of the most sensitive targets for oxidative stress. Endothelial activation caused by ROS exposure is a feature of several dysfunctions including diabetes, vascular toxicity, hypertension, ischemia, inflammation, acute and chronic tissue injury, atherosclerosis and hypertension [32–35]. Some of the genes modulated by ROS in these disease scenarios are endothelin-1 [23], pro-inflammatory mediators such as IL-6 [24] and vascular remodeling factors such as collagen 1 [25,26]. In agreement with these studies, we found these genes overexpressed in portal vein from cirrhotic rats together with MMP-2, TIMP-1, TIMP-2 and PlGF. The expression profile found in the immortalized PVECs from cirrhotic rats was quantitatively similar compared to whole portal vein tissue. The only difference was found in the expression of the MMP-2 gene, which was decreased in CH-PVEC, compared to CT-PVEC. This discrepancy could be attributed to the fact that whole portal vein extracts contain mRNA from other cell types, apart from endothelial cells, that may contribute to the overall mRNA expression differences. Despite this punctual difference, the use of iPVECs in our study has proven to be useful for the study of the portal vein abnormalities occurring in cirrhosis and, additionally, may provide us with basic scientific understanding of the mechanism leading to endothelial activation. For instance, the similarity found when comparing the iPVECs and the portal vein transcriptomes suggests that there may be an epigenetic component that maintains the differential expression despite the elevated number of cellular passages needed to generate the immortalized PVECs. Yet being a suggestive hypothesis, we need further investigation to confirm this link. As a downside of using the iPVECs cell line, we have observed negligible eNOS gene expression in all the cellular clones established. This phenomenon has previously described by others in the context of later culture passages of endothelial cells [36]. Therefore, and despite that the iPVECs still conserved other endothelial cells markers, some endothelial functions may be lost in the iPVECS. This drawback should be kept in mind when interpreting the data obtained in this cellular model of portal vein endothelium.

CeO_2_NP has caught considerable attention as a potential therapeutic tool in the prevention and treatment of oxidative stress related diseases. This interest relies on the expected properties of this nanomaterial to scavenge most of the ROS due to its multi-enzyme mimetic activities that resemble the mechanism of SOD, catalase and peroxidase enzymes [37,38]. The beneficial effects of CeO_2_ treatment have been reported in the fields of neurology [39–41], diabetes [42,43], retinal diseases [44,45], chronic inflammation [38,46], gastrointestinal epithelium inflammation [47], hepatoprotection against monocrotaline [48], liver cirrhosis [20] and cancer [47,49].

Consistently with these publications, we demonstrated that CeO_2_NPs treatment was effective in reducing the proinflammatory state of endothelial cells from the portal vein of cirrhotic rats. The main feature of this beneficial effect was the downregulation of IL-6 expression in both cultured PVEC and portal vein from cirrhotic rats. The similar responses observed in the *in vitro* studies with isolated endothelial cells and the *in vivo* models seem to indicate of the efficient targeting of the CeO_2_NPs to the cells of interest after i.v. administration. One of the potential explanations of these results is that the structural alterations in blood vessels occurring during inflammation enhanced CeO_2_NPs vascular uptake. Increased ROS contribute to vascular injury by promoting abnormal vascular cell growth, disruption of inner endothelial cell monolayer integrity and extracellular matrix remodelling [50,51]. These vascular abnormalities can be summarized as an increased roughness of the inner vascular layer, loss of the cobblestone endothelial structure and increased extracellular cell adhesion to the vasculature, in contrast to the uniform endothelial lining characteristic of the portal vein from control rats. Interestingly, the treatment of cirrhotic rats with CeO_2_NPs resulted in the correction of these morphological alterations. Despite that CeO_2_NPs flow modes in the blood stream are still a subject of debate [52,53], it is reasonable to hypothesize that CeO_2_NPs will be preferentially retained in rough and porous surfaces rather than in smooth and tight ones, promoting thus the passive targeting of CeO_2_NPs in the region of interest.

Many studies have reported high levels of the proinflammatory cytokine IL-6 in experimental models and patients with liver cirrhosis [54–58]. In addition, it has been proposed a link between high concentration of IL-6 and HCC [59,60]. Therefore, it is conceivable that the anti-inflammatory phenotype caused by CeO_2_ in the portal vein of cirrhotic rats could contribute to an improvement in liver function. We characterized further this anti-inflammatory effect by studying the polarization of the macrophages when they interact with CT-iPVECs or CH-iPVECs. The current model of macrophage polarization proposes two major populations of macrophages: M1 macrophages, which produce proinflammatory cytokines, ROS, which are prominently proinflammatory, and M2 macrophages, which promote tissue repair and stimulate vascular growth. M1-polarized macrophages express IL-1, TNF, IL-6, IL-12, IL-23, and iNOS in response to INF-γ; whereas M2 macrophages express IL-10, Decoy IL-1RII, IL-1ra and arginase in response to IL4. We showed that macrophages incubated with conditioned medium from CH-iPVEC were skewed to an M1 phenotype. In addition, the stimulation of macrophages with either IFN-γ or IL4 in the presence of conditioned medium from CH-PVEC potentiated M1 polarization and inhibited M2 polarization, respectively. Noteworthy, CH-iPVECs pre-treated with CeO_2_NPs lost their ability to potentiate M1 polarization while promoting a M2-like phenotype in macrophages. The implications of these results are encouraging. For instance, it has recently been reported that fibrosis improves with the injection of BM-derived macrophages. However it has not been determined whether macrophage polarization could influence the outcome of this treatment [55]. Therefore, it is reasonable to speculate that an anti-inflammatory/remodeling phenotype of therapeutic macrophages would be beneficial, given that exacerbated inflammation contributes to the perpetuation of liver damage. In this context, the results of our study suggest that the combination of CeO_2_NPs with cell therapy is worth to evaluate in the context of chronic liver diseases.

Some of the advantages of using CeO_2_ nanomaterials over the traditional antioxidative drugs are: 1) the multi-enzyme mimetic activities of CeO_2_NPs, which targets several sources of ROS generation and 2) the continuous regeneration of the CeO_2_NPs catalytic activity, which avoids the exhaustion of its anti-oxidative properties [61]. However, a big effort in standardization is still needed for the field to progress. In contrast to the soluble drugs used in clinics for which optimal dosages are determined in traditional toxicology studies, the therapeutic efficacy of the CeO_2_NPs does not depend exclusively of the dose. We should also consider other properties such as shape, density, surface area, size and physical-chemical properties of the nanoparticle in solution. Changes in these features may affect the catalytic activity and solubility of the nanoparticle [62,63]. Therefore, these parameters have to be properly tuned and standardized among different laboratories before this therapeutic strategy can be translated into the clinical area.

## Supporting information

S1 Fig Characterization of CH-PVEC gene expression pattern after CeO_2_NPs treatment. CT-iPVECs and CH-iPVECs were incubated with vehicle or 1μg/mL CeO_2_NPs for 24h. Cells were lysed in trizol and mRNA levels of Col1A1, endothelin-1, TIMP1, PlGF and MMP2, were quantified by real-time PCR. mRNA levels are illustrated as fold change relative to HPRT mRNA levels (n = 5).
(TIF)

S1 File Gel raw images. VE-Cadherin gel raw images: upper image shows VE-Cadherin western blot and lower image the ponceau staining.
(TIF)

S2 File ARRIVE Guidelines checklist. Document that aims to improve experimental reporting and reproducibility of animal studies for purposes of post-publication data analysis and reproducibility.
(PDF)
