# Supplementary figures and images for: Functionalized cerium oxide nanoparticles mitigate the oxidative stress and pro-inflammatory activity associated to the portal vein endothelium of cirrhotic rats
Source: PLoS One. 2019 Jun 24;14(6):e0218716. doi: 10.1371/journal.pone.0218716 (PMC6590813; doi:10.1371/journal.pone.0218716)

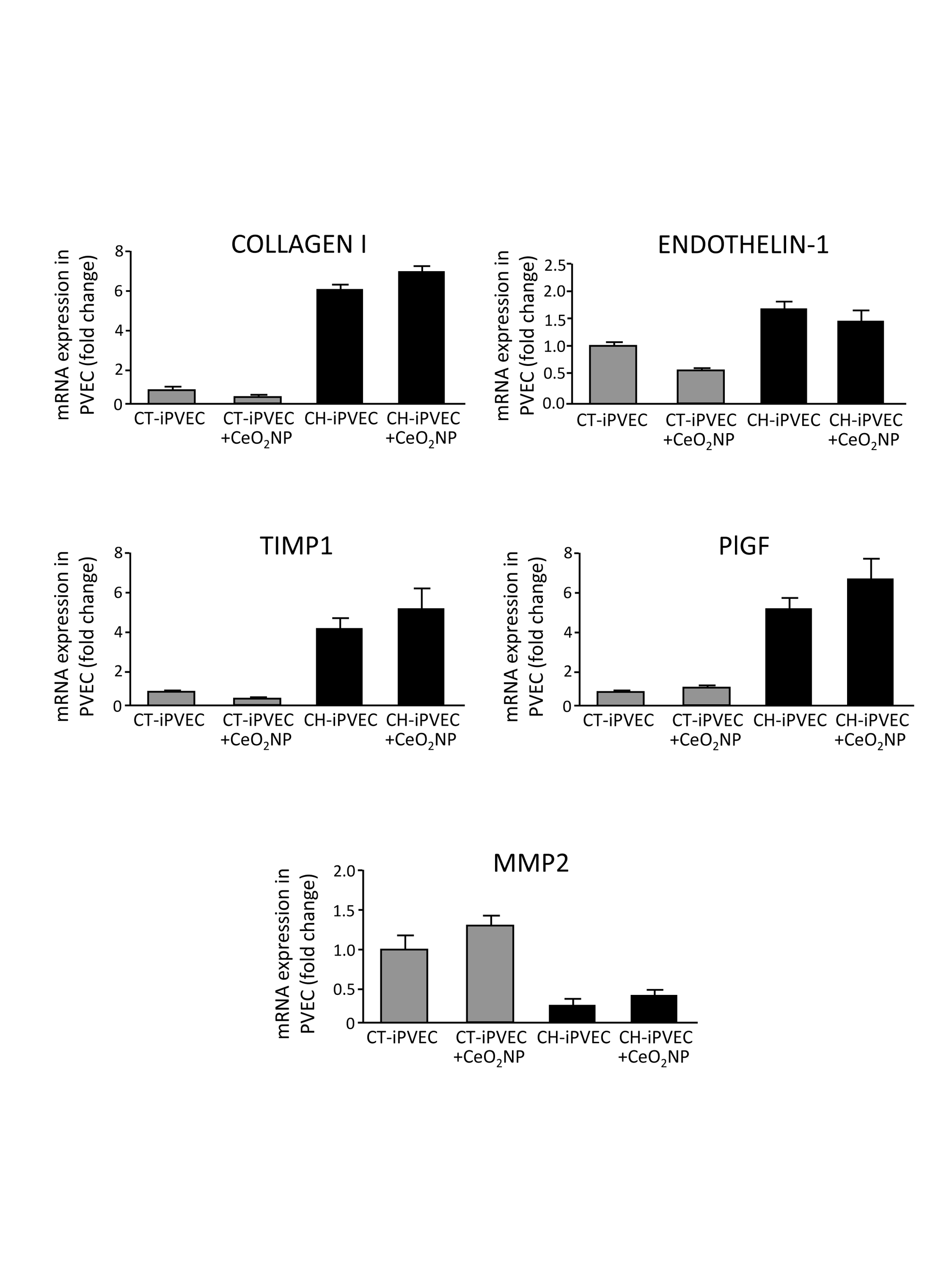

Supplement: S1 Fig — CT-iPVECs and CH-iPVECs were incubated with vehicle or 1μg/mL CeO2NPs for 24h. Cells were lysed in trizol and mRNA levels of Col1A1, endothelin-1, TIMP1, PlGF and MMP2, were quantified by real-time PCR. mRNA levels are illustrated as fold change relative to HPRT mRNA levels (n = 5). (TIF) [file pone.0218716.s001.tif]

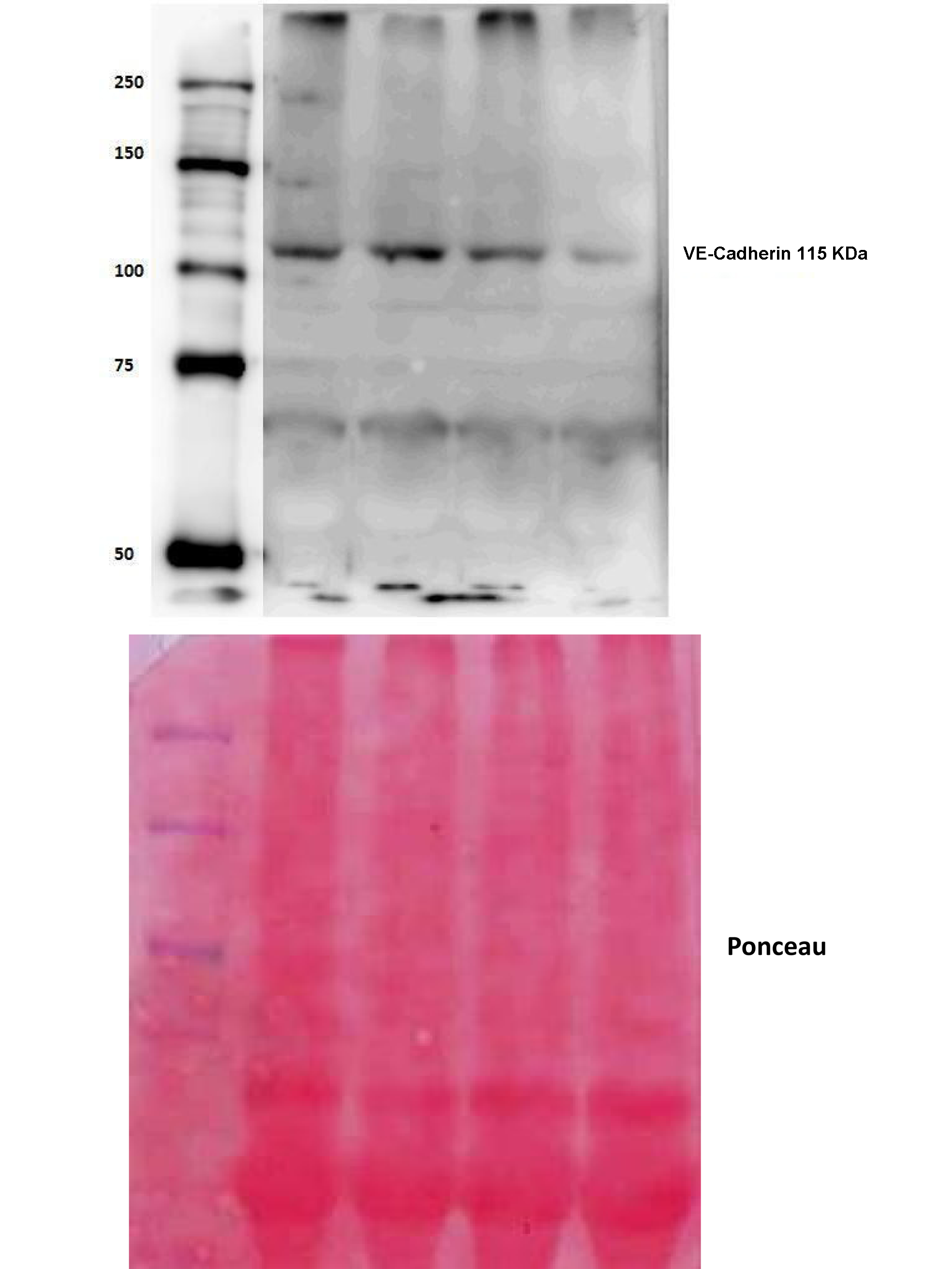

Supplement: S1 File — VE-Cadherin gel raw images: upper image shows VE-Cadherin western blot and lower image the ponceau staining. (TIF) [file pone.0218716.s002.tif]
